# The burden of neglected tropical diseases in Ethiopia, and opportunities for integrated control and elimination

**DOI:** 10.1186/1756-3305-5-240

**Published:** 2012-10-24

**Authors:** Kebede Deribe, Kadu Meribo, Teshome Gebre, Asrat Hailu, Ahmed Ali, Abraham Aseffa, Gail Davey

**Affiliations:** 1 Brighton and Sussex Medical School, Falmer, Brighton, United Kingdom; 2 Federal Ministry of Health Ethiopia, Addis Ababa, Ethiopia; 3 International Trachoma Initiative, The Task Force for Global Health, Addis Ababa, Ethiopia; 4 Faculty of Medicine, Addis Ababa University, Addis Ababa, Ethiopia; 5 School of Public Health, Addis Ababa University, Addis Ababa, Ethiopia; 6 Armauer Hansen Research Institute/ALERT, Addis Ababa, Ethiopia

**Keywords:** Names of WHO listed neglected tropical disease, Integration, Elimination, Ethiopia

## Abstract

**Background:**

Neglected tropical diseases (NTDs) are a group of chronic parasitic diseases and related conditions that are the most common diseases among the 2·7 billion people globally living on less than US$2 per day. In response to the growing challenge of NTDs, Ethiopia is preparing to launch a NTD Master Plan. The purpose of this review is to underscore the burden of NTDs in Ethiopia, highlight the state of current interventions, and suggest ways forward.

**Results:**

This review indicates that NTDs are significant public health problems in Ethiopia. From the analysis reported here, Ethiopia stands out for having the largest number of NTD cases following Nigeria and the Democratic Republic of Congo. Ethiopia is estimated to have the highest burden of trachoma, podoconiosis and cutaneous leishmaniasis in sub-Saharan Africa (SSA), the second highest burden in terms of ascariasis, leprosy and visceral leishmaniasis, and the third highest burden of hookworm. Infections such as schistosomiasis, trichuriasis, lymphatic filariasis and rabies are also common. A third of Ethiopians are infected with ascariasis, one quarter is infected with trichuriasis and one in eight Ethiopians lives with hookworm or is infected with trachoma. However, despite these high burdens of infection, the control of most NTDs in Ethiopia is in its infancy. In terms of NTD control achievements, Ethiopia reached the leprosy elimination target of 1 case/10,000 population in 1999. No cases of human African trypanosomiasis have been reported since 1984. Guinea worm eradication is in its final phase. The Onchocerciasis Control Program has been making steady progress since 2001. A national blindness survey was conducted in 2006 and the trachoma program has kicked off in some regions. Lymphatic Filariasis, podoconiosis and rabies mapping are underway.

**Conclusion:**

Ethiopia bears a significant burden of NTDs compared to other SSA countries. To achieve success in integrated control of NTDs, integrated mapping, rapid scale up of interventions and operational research into co implementation of intervention packages will be crucial.

## Review

### Background

The Neglected Tropical Diseases (NTDs) are a group of chronic parasitic diseases and related conditions that represent the most common illnesses of the world’s poorest people [1]. These diseases are the most common diseases of the 2·7 billion people globally who live on less than US$2 per day [2]. More than 1 billion people – a seventh of the world’s population – suffer from one or more Neglected Tropical Diseases [3]. Despite the substantial disease burden they impose, NTDs have largely been ignored in the global health architecture until recently. Social stigma, prejudice, marginalization and the extreme poverty of afflicted populations are among the factors contributing to the neglect of these diseases. Lack of funding for the prevention and treatment of these diseases is also a contributing factor [4].

Of more than seventeen NTDs, seven attract most attention because of their high prevalence and amenability to control worldwide [5]. These are the soil-transmitted helminth infections (hookworm, ascariasis, and trichuriasis); lymphatic filariasis; schistosomiasis; trachoma and onchocerciasis [5]. Globally, 600–800 million people have soil-transmitted helminth infections [5], 200 million people are infected with schistosomiasis, and 120 million with lymphatic filariasis in 83 countries [5,6]. Onchocerciasis affects nearly 37 million people in 34 countries, and is most abundant in Africa, with small foci in southern and Central America [7], while trachoma affects 84 million people globally [4].

NTDs have tremendous health and development impacts. These diseases hinder economic development, cause chronic life-long disability, and impair childhood development in the poor and disenfranchised communities in which they are most prevalent. They reduce child survival, educational attainment and agricultural productivity, and result in significant treatment costs [4,5,8,9].

In Ethiopia, most of the NTDs in the WHO list are present [10-25], except for probably dengue fever, Chagas disease and yaws. Although comprehensive, systematic and integrated responses are lacking, control programs for individual NTDs such as onchocerciasis and trachoma exist at national scale. Despite the huge burden of NTDs in Ethiopia, no comprehensive reviews have quantified the burden or distribution of these NTDs. This review was conducted to document the prevalence and burden of NTDs in Ethiopia.

We identified seminal articles published in peer-reviewed journals [10-37] and reports that were pertinent to the control of NTDs, using consultations with experts on this subject, and search of the key databases, including PubMed, archives of Ethiopian national journals and the WHO’s Weekly Epidemiological Record using as search terms the specific diseases listed as NTDs by the World Health Organization. The websites of central and regional governments and of international agencies were accessed for relevant reviews, guidelines, and databases. The exclusion and inclusion criteria for the papers were deliberately kept flexible. The scope of the review was increased on the basis of findings from the review of key papers and reports. Relevant published and unpublished technical documents were accessed for review. Senior experts in several NTDs were included to mediate between the information found in the literature and practical knowledge on the ground.

### Review of disease burden

#### Soil transmitted helminths

As indicated in Table 1 in Ethiopia, hookworm is estimated to infect 11 million people, thus Ethiopia bears 5.6% of the hookworm burden in Sub Saharan Africa (SSA) and is the country with the third highest burden in SSA [10]. Most parts of Ethiopia are suitable for the transmission of STHs, except parts of Somali and Afar regions where the annual mean temperature is too high for transmission [11]. The national prevalence of hookworm is estimated at 16% [12]. The prevalence of hookworm among school age children in Ethiopia was reported to be 38% in Jimma [13,14], 26.8% in Boloso Sore [15], 53% in central Ethiopia [16], 20.6% in Southwest Ethiopia [17], and 19% in northwest Ethiopia [18]. There was no significant gender difference [17]. According to a study conducted in southwest Ethiopia, 92% of the hookworm infections were due to *N*. *americanus* and 8% were due to *A*. *duodenale*. None of the cultures showed mixed infection (infection by two or more species) [13].

**Table 1:** **Summary of burden of neglected tropical disease in Ethiopia**, **2012**

**Disease**	**Geographical distribution**	**Burden of disease in Ethiopia**	**Proportion of SSA prevalence**[10]
Hookworm infection	Most of Ethiopia is suitable for transmission	11 million [10]	29%
Ascariasis	Most of Ethiopia is suitable for transmission	26 million [10]	25%
Trichuriasis	Most of Ethiopia is suitable for transmission	21 million [10]	24%
Schistosomiasis	Most of Ethiopia is suitable for transmission	5.01 million [19], 37.5 million at risk	25%
Lymphatic filariasis	Gambella (7), Beneshangul-Gumuz (13), SNNPR (9), Amhara (2) and Oromia (3) endemic districts. [20]	30 million at risk [10]	6%–9%
Onchocerciasis	Amhara Region (North Gondar), Benishangul-Gumuz (Metekel Zone), Oromia (Jimma, Illubabor, Wellega, West Shoa), SNNPR (Kaffa, Sheka and Bench Maji Zone) and Gambella.	5 million cases and 12 million at risk [12]	5%
Podoconiosis	One fifth of the surface of Ethiopia	1 million cases, 19.2 million at risk [21,22]	
Trachoma	Trachoma is found in all regions of Ethiopia. Six regions - Amhara, Oromia, SNNPR, Tigray, Somali and Gambella - bear high burden.	Ethiopia 10.3 million active trachoma, 1.3 million TT cases, [23], > 65 million at risk	3%
Human African trypanosomiasis	Historically Gambella and South Omo (SNNPR)	No cases of HAT since 1984 [24]	<0.01%
Leprosy	Leprosy has been reported from most part of the country except part of Afar and Somali region.	4,611 new cases per annum [25]	<0.01%
Leishmaniasis	VL is found in Tigray, Amhara, Oromia, Somali, Afar and SNNPR, whereas CL is prevalent in Tigray, Amhara, Addis Ababa, SNNPR, and Oromia.	4,000 new cases of VL per annum [26]20–50,000 cases of CL per annum [26]	<0.01%
Dracunculiasis	Gambella Region and historically South Omo (SNNPR)	8 cases in 2011 [27]	<0.01%
Buruli ulcer	Two case reported from Arbaminch Zuria district (SNNPR) and Tigray regions	2 cases reported [28,29]	<0.01%
Echinococcosis	Unknown	1817( 2.3/100,000) per annum [30]	Unknown
Rabies	Most part of the country	996-14694(12.6/million-18.6/100,000) per annum [31,32]	Unknown
Fascioliasis	Unknown	Unknown	Unknown

Ethiopia has the second highest burden of ascariasis in SSA: 26 million people are infected, which is 15% of the overall burden in SSA [10]. The prevalence among school age children was recorded at 28.9% in northern Ethiopia [33], 83.4% in southern Ethiopia [34], 22% in northwest Ethiopia [18], and the national average is estimated at 37% [12]. Similarly, Ethiopia has the 4^th^ highest burden of Trichuriasis, with 21 million people infected, which is 13% of the disease burden in SSA [10]. The national prevalence is estimated at 30% [12]. The global atlas of helminth infection (http://www.thiswormyworld.org/maps/ethiopia/archive) provides a predictive map of STH in Ethiopia.

#### Schistosomiasis

In Ethiopia, 5.01 million are thought to be infected with schistosomiasis and 37.5 million to be at risk [19]. The national schistosomiasis survey of 1988–89 reported an overall prevalence of 25% [35,36]. Among 365 communities surveyed for *S*. *mansoni* between 1961 and 1986, prevalence ranged from 10 to 92% [37]. Transmission occurs mainly through streams, irrigation schemes, and lakes. The intensity of infection correlates with severity of infection, and varies from locality to locality in Ethiopia.

In some studies the prevalence of *S*.*mansoni* infection was higher in children and adolescents [36], because children had higher environmental contamination potential. Prevalence in males and in females was 42.4% and 26.5% respectively [35].

#### Leishmaniasis

Ethiopia is one of the six countries (Bangladesh, Brazil, Ethiopia, India, Nepal and Sudan) in which more than 90% of global Visceral Leishmaniasis (VL) cases occur and one of the ten countries with the highest estimated case counts, which together account for 70 to 75% of global estimated VL incidence [1]. Both Cutaneous Leishmaniasis (CL) and VL are growing health problems in Ethiopia, with endemic areas that are continually spreading. Geographically, VL is found in Tigray, Amhara, Oromia, Afar, Somali and SNNPR, whereas CL is prevalent in Tigray, Amhara, SNNPR, Addis Ababa and Oromia regions.

**Figure 1:**
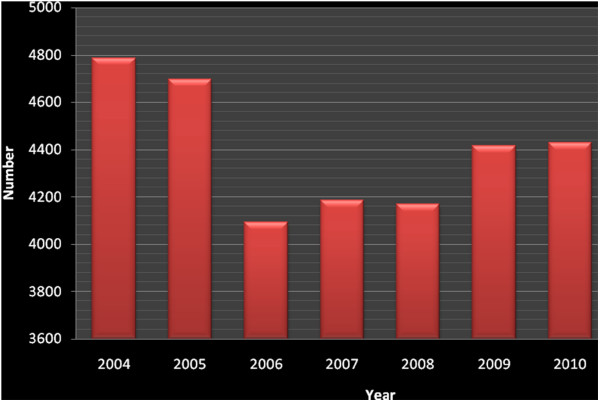
**Cases of Visceral Leishmaniasis,Ethiopia,2004-2010**[26]**.**

Historically the first case of VL in Ethiopia was identified in 1942 in southern Ethiopia. Every year, an estimated 3700–7400 cases occur in Ethiopia (Figure 1) [38]. The disease occurs in the lowlands of the northwest, central, south and southwestern parts of the country. In the north, the vector is associated with Acacia-Balanites forest, in the south with termite hills. In Ethiopia, VL affects mainly children and young adults (the mean age of affected in northern Ethiopia is 23) in endemic areas the mean age is much lower [26,38]. In northwest Ethiopia, where migrant laborers are at risk of exposure to VL, annual incidence ranges from 5 to 8 cases per 1000. The annual incidence among at risk populations in southern and south eastern Ethiopia ranges from 1 to 5 per thousand with huge geographical variation (AH unpublished observations).

CL has been well known since 1913, and is endemic in most regions, mainly in the highlands of Ethiopia in the altitude ranges of 1400 – 2900 m. It is a highly neglected disease with a zoonotic cycle involving rock hyraxes. There are estimated 20–50,000 cases yearly, but only 450 cases were reported in 2008 [26]. There are three clinical forms of CL in Ethiopia: localized CL, mucosal leishmaniasis and diffuse cutaneous leishmaniasis (DCL), all mainly caused by *L*. *aethiopica*. CL is most common in children [39,40]. In highly endemic areas, children less than 10 are affected, for example 8.5% of under 10s in Ochollo, southwestern Ethiopia [41]. The prevalence of CL in the vast majority of the endemic areas varies from 0.1% to 1.0% (AH unpublished observations); and higher prevalence rates were reported in hyperendemic areas, e.g. 3.6-4.0% in Ochollo [41], and 4.8% in Silti Woreda [42].

Outbreaks of leishmaniasis have occurred in Ethiopia. Between 2005–2008, a documented outbreak of VL occurred in Amhara Region (Libo Kemkem), with 2,500 cases and with a very high mortality [43,44]. An outbreak of CL occurred in Silti district 2003–2005 [45]. In 2010, cases of VL were identified in Tigray (Tahtay Adiabo district) and East lmey, a district in Somali region [38]. The incidence of HIV-Leishmania co-infection is very high (23% in 2008) in north Ethiopia [46,47].

#### Lymphatic filariasis

Lymphatic filariasis (LF) is a parasitic disease of man caused by three species of filarial parasites: *Wuchereria bancrofti*, *Brugia malayi* and *B*. *timori*, which are transmitted by anopheline and culicine mosquitoes [6,48,49]. LF is one of the most debilitating and disfiguring diseases in Ethiopia and is caused by *W*. *bancrofti*. The adult worms inhabit the lymphatics, and may lead to lymphoedema and elephantiasis. The disease is poverty-related and predominantly affects the poor and marginalized people [20,49]. In Ethiopia, 30 million people have been estimated to be at risk of LF, which would make Ethiopia the 4^th^ highest burden country in SSA, bearing 7.8% of the burden of LF in SSA (Table 2). However, some experts question the validity of this estimate, considering it to be an overestimation compared to recent surveys. The on-going mapping activities are intended to provide a realistic figure about the numbers of people at risk.

**Table 2:** **Burden of neglected tropical disease in Ethiopia and relative contribution and rank within Sub**-**Saharan Africa**, **2012**

**Disease**	**Ethiopia**	**SSA**[10]	**Percentage contribution of Ethiopia to SSA**	**Disease burden rank from SSA**
Hookworm infection	11 million [10]	198million	5.6%	3
Ascariasis	26 million [10]	173 million	15.0%	2
Trichuriasis	21 million [10]	162 million	13.0%	4
Schistosomiasis	5.01 million [19]	192 million	2.6%	14
Lymphatic filariasis	Ethiopia 30 million at risk [10]	382–394 million at risk	7.6%-7.8%	4
Onchocerciasis	5milion , 12 million at risk [12]	37 million	8.1%	Using annual treatment figures provided by APOC in 2010 as proxy indicators, Ethiopia stands 4th following Nigeria, DRC and Cameroon.
Podoconiosis	1 million cases, 19.2 million at risk [21,22]	4 million	25%	1
Trachoma	Ethiopia 10.3 million [10,23]	30 million	34.3%	1
Human African trypanosomiasis	0 since 1984 [24]	50,000-70,000	0	
Leprosy	Ethiopia 4,611 annual [25]	30,055	15.3%	2
Leishmaniasis	Ethiopia 4,000 new Cases annual [26]	19,000–24,000	16.7%-21.1%	2
Dracunculiasis	8 cases in 2011 [27]	1058	0.75%	4
Buruli ulcer	2 cases [28,29]	> 4,000	Unknown	Unknown
Fascioliasis	Few cases reported	Unknown	Unknown	Unknown
Echinococcosis	1,817annual [30]	Unknown	Unknown	Unknown
Rabies	996-14694 annual [31,32]	Unknown	Unknown	Unknown

According to recent mapping based on 11,685 individuals living in 125 villages (112 districts) of western Ethiopia, the prevalence was 3.7%, but high geographical clustering and variation in prevalence (ranging from 0% to more than 50%) was found. The prevalence of hydrocele (in males) and limb lymphoedema was 0.8% and 3.6%, respectively. Endemic districts were identified in the following regions: Gambella Region (seven districts), Beneshangul-Gumuz Region (thirteen districts), and Southern Nations, Nationalities and Peoples’ Region (SNNPR) (nine districts). The other five districts were from Amhara (two districts) and Oromia (three districts) regions [20].

#### Podoconiosis

Podoconiosis (endemic non-filarial elephantiasis) is a non-infectious geochemical disease caused by exposure of bare feet to red clay soil derived from volcanic rocks. Ethiopia is estimated to bear one fourth (25%) of the global burden of podoconiosis, with up to 1 million cases of podoconiosis existing in Ethiopia [21,22,50]. The disease occurs in highland red clay soil areas, mainly among poor, barefoot agricultural communities, who do not wear protective shoes. In endemic areas of Ethiopia, the prevalence of podoconiosis is high – 9.1% in Illubabor Zone, Oromia Region [51]. The socio-economic impact of the disease is high: of 10 patients, seven to nine tend to belong to the economically active age group population, and podoconiosis is estimated to result in a loss of USD 1.6 million per year in one zone of 1.6 million people alone, suggesting that at national level, the economic losses due to podoconiosis may be higher than USD 200 million per year [22]. Podoconiosis is also one of the most stigmatizing diseases in endemic areas. The disease leads to social exclusion of individuals and their families [52].

#### Trachoma

Trachoma, caused by *Chlamydia trachomatis* (an obligate intracellular bacterium), is the leading infectious cause of blindness worldwide [53].

The national blindness, low vision and trachoma survey conducted in Ethiopia in 2005/6 suggests that Ethiopia is the most trachoma-affected country in the world. The entire rural population of approximately 65 million people is at risk of blinding trachoma. It was estimated that in 2008, there were 9.84 million children with clinical signs of active disease and 1.36 million adults with trachomatous trichiasis. In the same study, projections suggested that in 2008 a total of 1,143,151 people were blind from avoidable causes, of which trachoma accounted for 11.5%. Provided appropriate interventions are in place, about 90% of all blindness in the country is avoidable [23,54]. The prevalence of blindness in Ethiopia is thought to be the highest in the world. After cataract, the preventable bacterial infection trachoma was the second-leading cause of blindness in Ethiopia. There are 10 million individuals with active trachoma in the country placing the vast majority of the population at risk. The prevalence of active trachoma was 40.1% among children 1–9 years old [54,55]. Ethiopia ranks first in the list of high burden SSA countries and bears 34.5% of the trachoma burden in the region. Ethiopia is one of the five countries including Guinea, India, Nigeria and Sudan bearing half of the global burden of active trachoma [1].

Trachoma is widely distributed in Ethiopia, with six regions bearing high burdens namely Amhara, Oromia, SNNPR, Tigray, Somali and Gambella regions [55].

#### Onchocerciasis

Onchocerciasis, also known as river blindness, is caused by a nematode filarial worm, *Onchocerca volvulus* that causes blindness and debilitating skin lesions [56].

The existence of onchocerciasis in Ethiopia has been known since 1939 as a result of investigation by Italians in south-western Ethiopia [57]. In Ethiopia, 5 million are estimated to be infected, with a further 12 million at risk from this disease [12,58,59]. The recent REMO mapping activity estimated that 5.2 million people are living in hyper- or meso-endemic areas [60]. Prevalence of onchocerciasis varies from place to place, from 84% in western endemic areas [61], to 19.5% in the northwest [19].

Onchocerciasis in Ethiopia is confined to the western part of the country, despite the presence of the vector in the other parts of the country. APOC-sponsored, nation-wide Rapid Epidemiological Mapping of Onchocerciasis (REMO) was first conducted in 1998. As a result, onchocerciasis was found to be prevalent in the North Gondar zone (Amhara Region), in Metekel and Assosa zones (Benishangul Region), Agnua and Mezhenger zones (Gambella region), in Illubabor, Jimma, East and West Welega zones (Oromia region), and in north Omo, South Omo, Kaffa Sheka and Bench-Maji zones (SNNPR [61]. In 1999 the National Onchocerciasis Control Program was established. The National Onchocerciasis Task Force (NOTF) was established in 2000 and the first CDTI project was launched in Kaffa-Sheka Zone in the same year. REMO refinement surveys were conducted in 2001, 2004 and 2011 [62].

#### Leprosy

In 2010, Ethiopia was one of the 17 countries reporting 1000 or more new cases per annum. Between 2004 and 2010, 4000–4500 new cases were diagnosed at health facilities annually. Ethiopia is the second highest burden country in SSA, after the Democratic Republic of Congo [25,63]. However, according to WHO, Ethiopia reached the leprosy elimination target of 1 case/10,000 population in 1999, and since then, the incidence has not changed appreciably [64]. As in other endemic countries, about 5,000 new cases are detected yearly and over 30,000 people are living with permanent leprosy-related disability. In 2002, clusters of endemicity with prevalence rates higher than the elimination target were recorded in four of the 14 administrative regions in the country [64]. In 2010, the total number of leprosy patients registered in the country was 5,303, and of these, 4,430 were new cases. Of the registered new cases, 1,308 were female and 331 children. In the same year, 357 relapse cases were registered [63].

**Figure 2:**
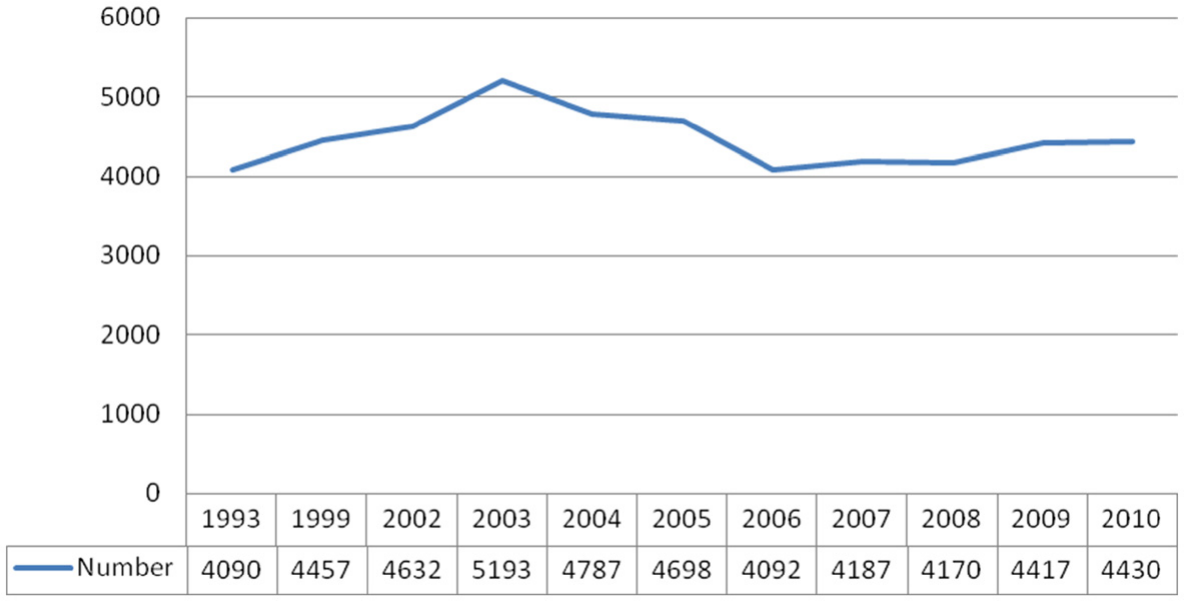
**New cases of leprosy,Ethiopia,1993–2010.**

Ethiopia ranked 7th among the 18 countries that report 93% of all new cases detected globally in 2009, although prevalence dropped from 5081 to 4516, the average number of new cases remained constant at around a mean of 4524 (range 4153–4940) between 2001–2011 (Figure 2). This translated to a drop in national case notification rate of 0.8/10,000 to 0.6/10,000. A 7.8% proportion of children under 15 and prevalence of 9.8% grade II disability rate among those newly diagnosed suggests an unknown magnitude of hidden cases. Regional variation in case notification rate varied between 0.16/10,000 in SNNPR (which nevertheless had a grade II disability rate of 45%) to 0.76/10,000 in Oromia [65].

#### Rabies

Rabies is an important neglected zoonotic disease. Annually 996–14,694 cases of human rabies are estimated to occur in Ethiopia [31,32], mostly acquired through dog bites [66-68]. According to a study in and around Addis Ababa, 92% of humans who received post-exposure anti-rabies treatment had been bitten by dogs. In Africa, the highest recorded number of human deaths due to rabies for the year 1998 was 43, reported from Ethiopia [69]. Most cases of fatal rabies occur among children in Ethiopia [69]. Almost all of these deaths are preventable through prompt medical attention comprising wound cleaning and post-exposure prophylaxis with rabies vaccine. Often all had attempted some form of herbal remedies by traditional healers before presenting to health facilities [69]. There has been no apparent decline in the number of recorded human rabies cases over 20 years [69,70].

#### Dracunculiasis (guinea worm)

Dracunculiasis is caused by the parasitic filarial worm *Dracunculus medinensis*, the largest of all the filarial worms (nematodes) affecting human [71,72]. Dracunculiasis used to be a formidable public health problem, mainly in terms of morbidity, incapacity and suffering of those affected. About 50% of cases suffer from secondary infections and become severely incapacitated [73].

**Figure 3:**
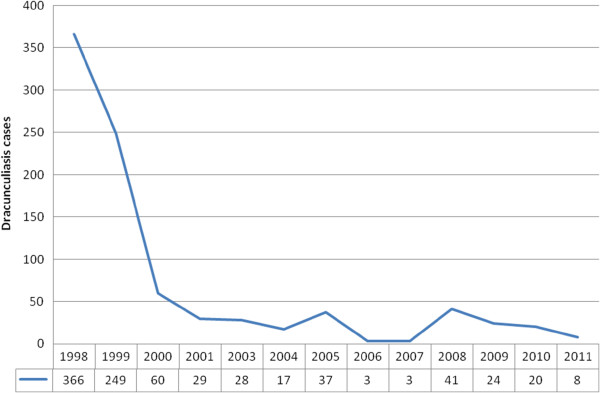
**New cases of Dracunculiasis,Ethiopia,1998–2011.**

In Ethiopia a case of dracunculiasis was reported first in 1969 [74]. Geographically, the disease was prevalent in Gambella region and South Omo (SNNPR). The eradication program in Ethiopia stated in 1990, and has reduced the number of cases from 1,252 in 1994 to only 8 in 2011(Figure 3) [27,75,76]. Ethiopia is one of the four countries that reported dracunculiasis in 2011. The key challenge to achieving complete interruption of transmission is the very frequent migration and interaction of the people along the Ethio-Sudan border and very high likelihood of cross-border cases from South Sudan.

#### Other NTDs in Ethiopia

Human African trypanosomiasis has previously been reported in Ethiopia. The geographical distribution was in Gambella, with sporadic cases reported from Gamo Gofa, Keffa and Wellega. Since 1984 there have been no cases reported to WHO [24,75,77].

Echinococcosis is a zoonotic disease caused by four species of *Echinococcus*: *E*. *granulosus* (causing cystic or unilocular echinococcosis); *E*. *multilocularis*, *E*. *vogeli* and *E*. *oligarthrus* (species causing polycystic or alveolar echinococcosis). In Ethiopia, humans become infected accidentally with *E*. *granulosus* through contact with dog’s feces [75]. In a review of 36,402 patients admitted for ultrasound examination, an incidence of 2.3 cases per 100,000 per year was estimated [30].

Buruli ulcer is caused by infection with *Mycobacterium ulcerans*. In Ethiopia, only 2 cases were reported [28,29] from Arbaminch Zuria district. Ethiopia is not in the list of thirteen countries listed as endemic for Buruli ulcer in the African region.

### Current status of control and elimination of neglected tropical diseases in Ethiopia

#### Onchocerciasis

Although onchocerciasis was reported as early as 1939, part of the country was recognized to be endemic only in the 1970s [57]. The first national plan to fight onchocerciasis was developed in 1999. In 2000, the National Onchocerciasis Task Force was established by Ethiopia's Ministry of Health with a mission to: mobilize and educate onchocerciasis-endemic communities; coordinate Mectizan® tablet procurement (donated by Merck) and distribution; and coordinate all partners in the program [57,62]. The Carter Center, Light for the World and the African Program for Onchocerciasis Control (APOC) play a critical role in supporting the Mectizan distribution program in these areas. The program went on to expand into other areas, doubling treatments each year until reaching scale in 2004. Treating more than 4 million annually, the program’s geographic coverage reached 99.2% and sustained therapeutic coverage of 77.4% (Table 3) [60].

**Table 3:** **Onchocerciasis treatment in CDTI zones in 2010 in Ethiopia [**[62]**]**

**CDTI Zone**	**Ultimate treatment goal**	**Total population**	**Population treated 2010**	**% Ultimate treatment goal**	**Percent total population treated**
Kaffa	840,886	1001,055	784,716	93	78
Sheka	180,053	214,349	177,540	99	83
Bench Maji	579,848	690,295	543,038	94	79
North Gondar	238,369	283,773	215,632	90	76
Illubabor	648,750	772,321	639,544	99	83
Jimma	765,511	911,323	743,218	97	82
Metekel	127,079	151,284	121,072	95	80
Gambella	84,611	101,013	73,435	87	73
Total	3,465,107	4,125,413	3,298,195	95	80

#### Trachoma

Ethiopia has a two-phase national five year Strategic Plan for eye care. Since 2000, Ethiopia has been implementing the World Health Organization-approved SAFE strategy for trachoma control — surgeries, antibiotics, face and hand washing and environmental hygiene. Through The Carter Center alone, using what is known as the MalTra-Week Strategy (combining malaria case detection and treatment with mass azithromycin distribution) more than 14.7 million people received azithromycin in 2010 [62]. In 2011, a total of 17.7 million people were treated with azithromycin in 195 districts. Over the past few years, annually about 60 – 90,000 cases of trichiasis have received TT surgery (FMOH, unpublished annual reports). Hygiene education and latrine promotion has been implemented nationwide through the health extension program. As a result, it was confirmed by DHS 2011 that individual latrine coverage (ownership and utilization) has reached 45% for rural households [78].

A number of clinical trials and pieces of operational research have been conducted over several years, to guide effective implementation of interventions for the eventual control and elimination of trachoma in Ethiopia. A PubMed search indicated that a total of 86 trachoma research papers from Ethiopia have been published in local and international journals since 2001.

#### Dracunculiasis

The Ethiopian Ministry of Health established the National Dracunculiasis Eradication Program in 1993, and launched a village-by-village nationwide search during which 1,120 cases were found in 99 villages in the southwest part of the country [57,75]. Transmission of Guinea worm disease in the Southern Nations, Nationalities and Peoples Region (SNNPR) was interrupted in 2001 [75]. In 2007, Ethiopia reached a milestone by reporting zero indigenous cases for more than 12 consecutive months. Unfortunately, transmission of the disease resumed in 2008 when the country reported 41 indigenous cases [75]. The unexpected resurgence of Guinea worm disease in Gambella Region during 2008 demonstrates the constant need for vigilance in eradication efforts. Ethiopia reported 8 cases of dracunculiasis in 2011, including two cases imported from South Sudan [27]. In addition to the active case finding program, it is vital to enhance behavioral change and mobilize communities to prevent contamination of sources of drinking water. A cash reward system for case detection and reporting was found to be very helpful in facilitating effective surveillance and case containment activities.

#### Lymphatic filariasis

In 2009, with the support from The Carter Center, the Ethiopian Ministry of Health launched a LF elimination program in five districts of Gambella Region. The program reached 84% of its target by providing annual MDA of a single dose of ivermectin and albendazole to a target of almost 100,000 people. Recent LF mapping has identified new endemic districts in other regions indicating the need for expansion of the program to these places [20,62]. Integration must take into account both treatment goals and target group [79]. Currently there are other initiatives to establish sentinel sites and expand the treatment program.

#### Podoconiosis

Community-based lymphoedema management for podoconiosis was started in 1998 in Ethiopia by a non-government organization in Wolaita. The treatment appears to be effective, and patients show improvement after an average of three months treatment [79], though rigorous controlled assessment of this treatment is still necessary. The treatment includes foot hygiene, bandaging and elevation. Currently it is being run in three Regions of Ethiopia [80,81]. Although the implementation is still at small scale, it is attracting the attention of health care providers and health authorities for future possible integration into the national health system.

#### Soil transmitted helminths

Ethiopia launched an Enhanced Outreach Strategy (EOS) in 2004; one of the objectives of the initiative was to deworm 2–5 year old children every six months. The strategy was implemented in every district in the country except Addis Ababa and by 2009; the program had reached more than 11 million children in 624 districts. Every six months, with UNICEF support, Regional Health Bureaus organize the EOS. Each district has one EOS team per sub-district, composed of one health worker and one HEW who mobilize the community to come to the nearest health post on a specific day, the EOS day, when the EOS team deworms all children under five years and distributes vitamin A supplements. In many instances, the Regional Health Bureaus use this opportunity to deliver other essential services, such as measles vaccination, tetanus vaccination, mosquito net distribution, HIV/AIDS prevention, or iodine capsule distribution [82].

#### Schistosomiasis

Although Ethiopia is highly endemic for schistosomiasis, control of this disease is still in its infancy, and no recent mapping of schistosomiasis has taken place [83]. At a stakeholders meeting convened in July 2012 by the Schistosomiasis Control Initiative and the Ministry of Health, nationwide mapping of schistosomiasis integrated with mapping of other NTDs was planned for 2013, and strategies to expand MDA on the basis of the mapping outlined.

#### Leishmaniasis

In 2006, a leishmaniasis control program that included mandatory notification was established. Although patients are not required to pay for VL drugs and rK39 tests, other tests are not free of charge. It is estimated that treatment of VL patients usually requires a high cost to complete a full course of antimony-based treatments [26], and many are too poor to pay for these services. There are no vector control programs in place specifically for leishmaniasis, and bed net distribution and insecticide spraying take place in the context of malaria control [26,38,39]. A national Leishmaniasis control guideline has been developed. A geographic information system (GIS)-based risk mapping of leishmaniasis is being completed for the country and treatment guidelines for cutaneous leishmaniasis have been developed through an international consultation process organized by the FMoH in collaboration with the Armauer Hansen Research Institute (AHRI) and the World Health Organization (Proceedings available at AHRI).

#### Leprosy

An organized leprosy control program was established within the Ministry of Health in 1956 with a detailed policy issued in 1969 operating as a vertical program. Multiple Drug Therapy (MDT) was implemented in 1983 leading to relatively rapid reduction in prevalence of leprosy. In 1994, leprosy was combined with tuberculosis under a joint control programme. By 2001, the leprosy component had been fully integrated into the general health services [65].

The FMoH is pursuing an Enhanced Global Strategy of integration with the general health service, reaching undeserved communities and effective partnership to reduce the rate of new cases with Grade II disabilities by at least 35% by the end of 2015, compared to the baseline at the end of 2010 in line with the HSDP target of reducing Grade II disability to 1% by 2015 [65].

Ethiopia achieved the leprosy elimination target of 1 case/10,000 population in 1999. The leprosy control program has been integrated with the tuberculosis control program within the national health system. Diagnosis and treatment services are provided free of charge for patients in every health center. In addition, rehabilitation services are provided for patients [63,64]. Early case detection remains a critical challenge. Specialized Leprosy expertise at the central level has been depleted over the last few years because of the shift of funding to tuberculosis and other diseases and resulting drift in brain flow.

#### Other neglected tropical diseases

In Ethiopia there have been no reported cases of HAT since 1984 [24]. No national program for control of rabies exists, although a national rabies survey is underway and there are sporadic initiatives to vaccinate dogs and provide post exposure vaccination free of charge. There is currently no detailed information about the extent of Buruli ulcer, echinococcosis and fascioliasis.

## Discussion

This review indicates that Neglected Tropical Diseases are significant public health problems in Ethiopia. Compared to other countries in sub-Saharan Africa, Ethiopia bears a significant burden of many of these diseases. However, disease burden estimations are based on limited and often old data. From the analysis reported here, Ethiopia stands out as having the third largest total number of NTD cases following Nigeria and DRC. Ethiopia is estimated to have the highest burden of trachoma, podoconiosis and cutaneous leishmaniasis in SSA, the second highest burden of ascariasis, leprosy and visceral leishmaniasis, and the third highest burden of hookworm. Infections such as schistosomiasis, trichuriasis, LF and rabies are also common, yet despite these high burden infections, the control of most NTDs in Ethiopia is very limited.

Understanding which geographical areas require intervention is fundamental for cost-effective disease control [79,84,85]. Mapping of diseases should be preceded by review of existing data and followed by collection of data for those areas lacking this information. The most recent Rapid Epidemiological Mapping of Onchocerciasis identified new foci of transmission (meso and hyper endemic communities) that require mass treatment with Ivermectin [62], as well as areas to be refined before final decisions over inclusion or exclusion from treatment. Similarly, the western part of Ethiopia was mapped for Lymphatic Filariasis [20] and identified new transmission foci of LF beyond the previous altitude limits of transmission. Further mapping is therefore necessary to build a complete picture of the geographical distribution of LF in Ethiopia. Spatial analysis of a map compiling historical and recent data on podoconiosis distribution [86] indicated the presence of large scale spatial trends in the distribution of podoconiosis [Deribe K, Brooker SJ, Pullan RL, Davey G: *Spatial distribution of Podoconiosis in Ethiopia: Results from historical maps and their implication on contemporary disease control*.; unpublished], but generated insufficient evidence to classify areas as endemic or non-endemic for podoconiosis. Collection of data for mapping of podoconiosis will improve understanding of the spatial distribution of podoconiosis and ecological factors determining this distribution. Experiences from Uganda [79,87], Togo [88] and South Sudan [89] indicate the possibility of integrated disease mapping [90]. Togo and South Sudan conducted integrated mapping of STH, LF, trachoma, schistosomiasis, and onchocerciasis. These surveys were found to be cost-effective [89], with commendable epidemiological rigor. Such integrated disease mapping will have implications both in efficient resource utilization and integrated disease control. For example, integrated mapping of LF and podoconiosis is possible: the large scale autocorrelation of podoconiosis [Deribe K, Brooker SJ, Pullan RL, Davey G: *Spatial distribution of Podoconiosis in Ethiopia: Results from historical maps and their implication on contemporary disease control*.; unpublished] suggests that sample sizes designed for LF will be more than adequate to capture the spatial distribution of podoconiosis. Second, diagnosis of LF needs exclusion of podoconiosis and vice versa, hence integrating the mapping of these two diseases will bring benefits in terms of reduced costs.

In Ethiopia, the nationwide blindness and trachoma prevalence survey conducted in 2006 [54] was followed by implementation of a five year strategic plan. To monitor the progress of this plan and identify areas that require further intervention, it will be necessary to update the trachoma map. Experience from Ethiopia has shown the feasibility of integrating trachoma surveys with malaria surveys, resulting in reduced costs, although some logistical challenges may arise [91].

It is not always mandatory to conduct surveys for mapping. Historical data modeled for environmental and demographic changes may be used for mapping the spatial distribution of disease and identifying populations at risk. For example in Kenya [92], historical and contemporary survey data were used to guide disease control. In Ethiopia many surveys have been conducted on soil-transmitted helminth (STH) infections and schistosomiasis [11,37], and these might be used to identify high risk areas for prioritization and generate maps for initiating interventions, as appropriate.

**Figure 4:**
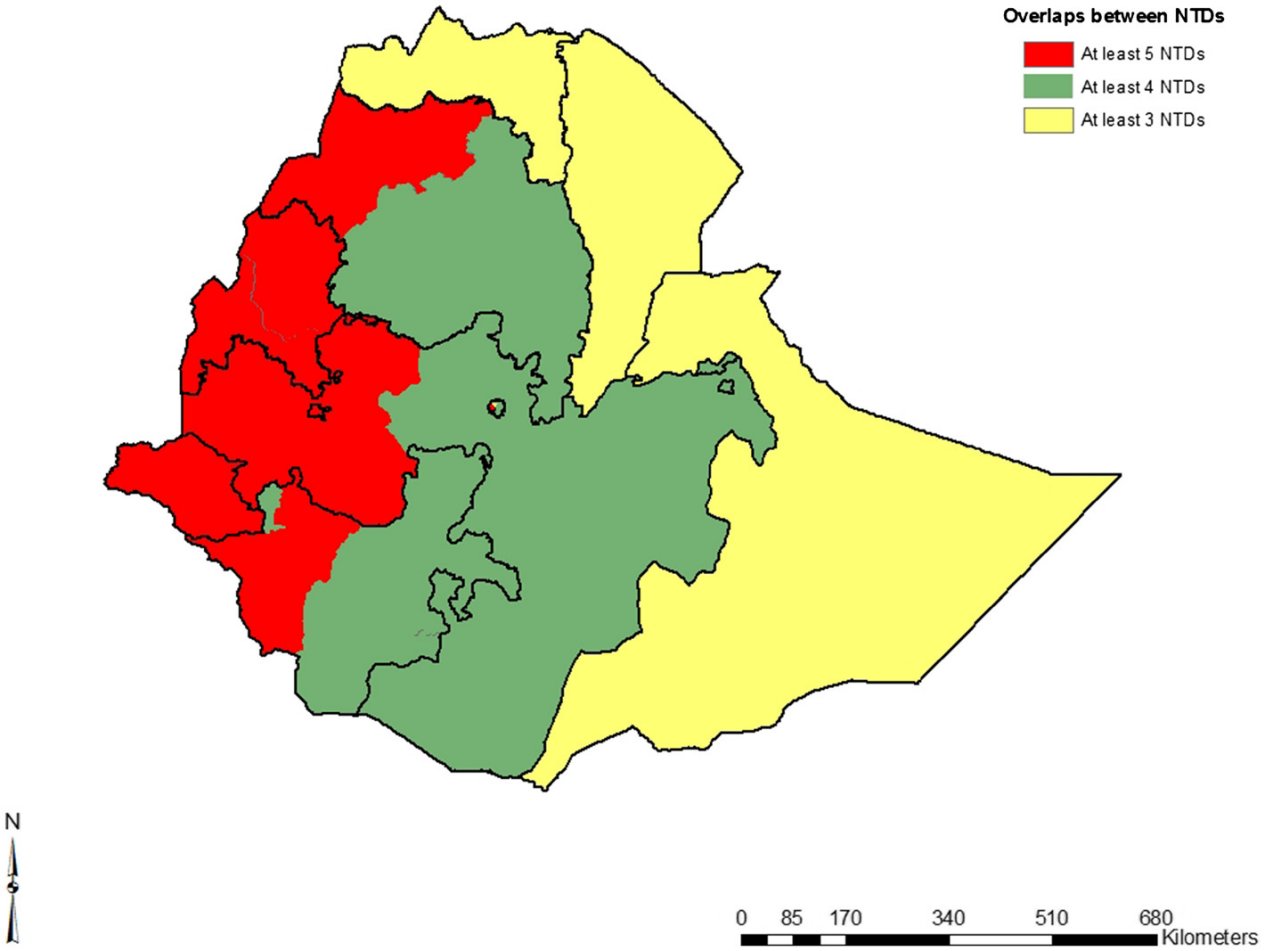
**Overlap between five common NTDs(soil**-**transmitted helminth infections;lymphatic filariasis;schistosomiasis;trachoma and onchocerciasis)in Ethiopia as reported by health providers and maps.**

Traditional efforts to treat and prevent NTDs through vertical programs are often costly, and the integration of program components has the potential to cut the costs of NTD programs [93-97]. Because NTDs tend to overlap in geographic areas (Figure 4), it is logical to attempt an integrated approach to NTD control [93]. Since 2004, there has been greater advocacy for the integrated control of NTDs [94]. In Ethiopia there are geographical overlaps among NTDs, for example, according to the recent mapping of LF in Ethiopia [20], overlap between LF and onchocerciasis occurs in considerable geographical areas in the southwest of the country. Out of 34 LF endemic districts, 20 were also endemic for onchocerciasis. The existence of a well-established onchocerciasis control program in Ethiopia suggests that integration of other NTDs into this program might successfully build on the existing networks of community based drug distributors (CDDs). In addition, a successful trachoma prevention and treatment program exists, into which MDA and deworming campaigns might be integrated. One practical example is the integration of trachoma services into the existing onchocerciasis control program through Community Drug Distributors in North Gondar. In most of the Community Directed Treatment with Ivermectin (CDTI) areas, the malaria program is integrated into the daily activities of CDDs. Malaria prevention activities are now included in the integrated CDD training course. CDDs are trained to record the number and condition of long lasting insecticidal nets (LLIN) [62]. Prevention efforts such as shoe wearing for podoconiosis may also help in prevention of chronic larva migrans and snakebite, so health promotion emphasizing the multiple benefits of shoe wearing may be valuable.

The high prevalence of HIV-leishmaniasis co-infection in Ethiopia [45,46] brings an opportunity to integrate VL treatment and care with existing HIV treatment and care. While providing leishmaniasis patients with the option for HIV counseling and testing, screening of HIV positive individuals for leishmaniasis in endemic areas may avoid missed diagnoses. Hotez and colleagues [98] argue that the integration of NTD control may accelerate reductions in the prevalence or severity of HIV/AIDS, tuberculosis, and malaria.

Integration of NTD control into the existing health system is also another important issue. The integration of NTD control into the heath system will help ensure the sustainability of programs by sharing resources with better resourced control programs such as HIV/AIDS, tuberculosis and malaria [99]. In Ethiopia the control of NTDs is coordinated by a focal person in the Federal Ministry of Health, and the existence of more than 38,000 trained Health Extension Workers (female salaried health workers) offers great opportunities for integrated NTD control. Two health extension workers are located in each *kebele* (the lowest administrative unit, consisting of approximately 1000 households). Possible areas of integration might include MDA, health education and hygiene promotion.

Health education is the principal component of most of the control and elimination programs. The target audiences of messages concerning NTDs are similar people, therefore identification of standard messages and production of materials which address multiple NTDs should be considered to harmonize the key NTD messages. Qualitative assessments and socio-cultural studies of knowledge, attitude and practice of endemic communities with respect to NTDs must be utilized in the development of key educational messages.

The other important area for integration is monitoring and evaluation of control and elimination activities. The current Health Management Information System (HMIS) in Ethiopia captures only a few of the NTDs, and unless this is rectified so that routine data on NTDs are collected via the health system, costly and inefficient surveys will be necessary to monitor NTD programs. Data collected via the HMIS could in the future benefit district offices as they monitor their own activities and make decisions at local level. It is anticipated that the National Master Plan for Integrated Control of NTDs 2012–2015 will outline a more precise road map for the implementation of integrated mapping and control of NTDs (Table 4).

**Table 4:** **Summary of WHO recommended control strategies and their status in Ethiopia**, **2012**

**Disease**	**National Target**	**WHO recommended control strategy**	**Status in Ethiopia**
Soil Transmitted Helminthes (STH) (Hookworm, Ascariasis and Trichuriasis)	To reduce morbidity due to STH to a level where it is no longer of public health significance.	Annual mass treatment of school age children and whole communities in high-prevalence areas	Deworming 2–5 year old children every six months nationwide
Schistosomiasis	To reduce morbidity due to schistosomiasis to a level where it is no longer of public health significance.	Annual mass treatment of school age children and whole communities in high-prevalence areas	No active control program case management and MDA in few places
Lymphatic filariasis	To eliminate LF as a public health problem by 2020	Annual MDA to treat the entire population for a (currently undefined) period, to interrupt transmission	Annual MDA in identified endemic areas since 2009
Onchocerciasis	To eliminate onchocerciasis as a public health problem by 2015	Vector control through spraying of larvicides and annual CDTI	CDTI since 2000
Podoconiosis	To control podoconiosis in Ethiopia	Under development; includes community-based treatment of cases consisting of foot hygiene, use of shoes, wound care, etc.	Community-based treatment of cases consisting of foot hygiene, use of shoes, wound care in few endemic places
Trachoma	To eliminate blinding trachoma through SAFE strategy by 2020	Surgery, antibiotic therapy, facial cleanliness and environmental improvement (SAFE) strategy	Surgery, antibiotic therapy, facial cleanliness and environmental improvement (SAFE) strategy
Human African trypanosomiasis	Cases were not reported since 1984	Case detection and treatment. Vector control through spraying, traps and targets	None
Leprosy	Eliminated from Ethiopia	Multidrug therapy	Multidrug therapy, reduce disability, early case detection
Leishmaniasis	To control leishmaniasis in Ethiopia	Case detection and treatment and personal protection through use of mosquito nets	Case management in endemic areas
Dracunculiasis	Eradication of Guinea worm in Ethiopia with certification by the international commission by 2015	Active case detection and containment, provision of water supply, abate application and use of cloth and pipe filters	Active case detection and containment, provision of safe water supply, abate application and use of cloth and pipe filters
Buruli ulcer	No target	Case detection, treatment and surgery	Case management
Echinococcosis	No target	Case detection and treatment, regular deworming of dogs, providing health information and inspecting meat.	Case management
Rabies	No target	Controlling rabies in both wild and domestic animals; providing pre-exposure immunization to humans at occupational risk of contracting the disease; and on delivering post-exposure prophylaxis to potentially exposed patients	Post-exposure prophylaxis to potentially exposed patients.
Fascioliasis	No target	Preventive chemotherapy and case detection and treatment	Case management

## Conclusion

Most NTDs are highly prevalent in Ethiopia; resulting in enormous disease burdens compared even with other Sub-Saharan African countries. However, despite these high burdens of diseases, the control of most NTDs in Ethiopia is in its infancy, and mostly underfinanced.

The key to control of NTDs lies in understanding the geographical distribution of disease in a given country. At the time of this review, only a few NTDs have been adequately mapped in Ethiopia. This indicates the need for integrated mapping to better understand the distribution of particular diseases and areas of overlap for treatment and control. Resource mobilization for conducting integrated surveys should be prioritized. Once the mapping is completed and disease distribution is known, cost estimates for the control of common NTDs within Ethiopia will enable resource mobilization and guide donors and partners.

The development of a National Master Plan for Integrated Control of NTDs is a huge step forward. The overarching goal: “to accelerate integrated control of NTDs in Ethiopia so that NTDs won’t be public health problems by 2015” will require coordinated efforts among a range of partners. Operational research into an integrated approach for control of NTDs will also be vital. Financing the control of NTDs in Ethiopia will have huge implications not only for Ethiopia but also for the SSA region.

A national coordinating body of NTDs has been established, and several task forces for specific NTDs exist. Mapping of organizations working on NTDs and their disease interests and geographical coverage would benefit better coordination. Although national level coordination is a priority, regional level coordination should also be given emphasis.

National and international best practices in NTD control must guide the establishment and scale up of interventions. Drawing lessons from the successful and integrated programs for CDTI and trachoma control in Ethiopia is important. Strengthening the health system to respond to NTDs is critical; particularly training health providers prior to service and in-service to adequately treat the NTDs. Accessing diagnostic supplies and medicine for treatment are also important.

A three pronged roll-out package consisting of: laying the groundwork; rolling out an integrated program; and establishing effective management has been demonstrated to be effective in other settings [97], and would likely benefit Ethiopia if adopted.

### Disease specific recommendations

• Onchocerciasis: The CDTI strategy for the control of onchocerciasis has been very effective in reducing and, in some cases, halting transmission in known endemic foci as witnessed by APOC evaluation. Currently, the issue of moving from control to elimination of onchocerciasis in Ethiopia and elsewhere is under consideration by APOC and its partners. It will be crucial to conduct a complete independent review of the national control program and use findings to reorient the program and design elimination strategies. In addition to programmatic reviews, the evaluation should consider entomological, epidemiological and parasitological aspects of control interventions. As evidenced by the elimination programs in the Americas, the initiative is very demanding in terms of human, financial and logistical resources. Therefore, efforts must be made to secure full government commitment and approval before commencing to launching such highly demanding elimination projects.

• Trachoma: The current implementation of the full SAFE strategy for trachoma control is limited mainly to the Amhara region and some parts of SNNPR and Oromia. If Ethiopia is poised to achieve the GET2020 targets, the program needs to scale up aggressively to all affected regions and most particularly in Oromia (where there is the 2nd highest burden of disease in the country). In order to clear the huge backlog of trichiasis, special surgical strategies should be designed in collaboration with the respective regional health bureaus and their partners/donors.

• Dracunculiasis: The Ethiopian Dracunculiasis Eradication Program (EDEP) is working very hard to achieve complete interruption of GWD transmission by 2012. Due to the apparent risk of reintroduction of infection from South Sudan (which is the only neighboring endemic country), the program must stay vigilant along the common borders to detect and contain cases as they occur. Heightening public awareness and publicizing the cash reward system to the whole country (using all available communication channels) should remain top priority for the national program. The National Certification Committee should continue to work closely with the program to ensure proper documentation of program interventions and preparation of a Country Report for the International Certification Commission.

• Leishmaniasis: Ethiopian Cutaneous Leishmaniasis, caused by th*e species Leishmania aethiopica*, is a major health problem in the highlands (1400 - 1900m) of Ethiopia, and the cycle is maintained zoonotically by rock hyraxes. There are no sensitive laboratory-based diagnostic tests, and treatments are variably effective. Thus case detection suffers from unavailability of simple and sensitive tests as well as absence of safe and effective treatments. New tools for diagnosis and effective treatments are needed. Epidemiological mapping is currently underway. Mapping the geographical distribution of sandfly vectors (*P*. *longipes* and *P*. *pedifer*) and rock hyraxes (*Procavia capensis* and *Heterohyrax brucei*) will complement efforts to map the disease. The integration of cutaneous leishmaniasis into the mapping and control efforts of other NTDs will be a challenge, and caution is needed so as not to undermine leishmaniasis control efforts.

• Visceral leishmaniasis, which largely occurs in the Ethiopian lowlands including the rift valley regions, is co-endemic with malaria. There is no evidence of overlap with onchocerciasis and LF. Co-infection with HIV in north Ethiopia and among specific risk groups (migrant workers and military personnel) is an issue that deserves public health intervention. The treatment of HIV co-infected patients remains a challenge. Effective treatments for achieving initial cure and secondary prophylaxis for prevention of relapses are needed. The availability of rapid diagnostic tests, e.g. rk39 dipsticks, has offered the opportunity for enhanced VL case detection. Nonetheless, treatments for VL remain prohibitively expensive. Attempts to shorten the duration of treatment from the current 4 weeks (antimony-based treatments) or 17 days (paromomycin and antimony combinations) to less than 10 days need to be strengthened and enhanced. While VL transmission areas are fairly well known, the potential of the disease to spread to new localities needs to be acknowledged. Mapping the geographical distribution of sandfly vectors (mainly *Phelebotomus martini*, and *P*. *orientalis*) must be enhanced. New tools for detection of asymptomatic infections in humans are needed; and defining their role in transmission is a critical step towards designing an effective control strategy.

• Lymphatic filariasis: The recently started mapping efforts and MDA-based interventions must be enhanced and laid on a solid programmatic foundation possibly integrated with the onchocerciasis control programme. The MDA-programs must be complemented with efforts to monitor effectiveness of the interventions as well as the efficacy of treatments, i.e., ivermectin and albendazole. For this to happen, it is necessary to establish sentinel epidemiological sites for baseline assessment, monitoring impact of interventions and efficacy of drugs. A close supervision of the programs in place is a vital step for ensuring success.

• Schistosomiasis: Mapping the distribution of schistosomiasis is an important first step to establishing a national schistosomiasis control program. While the mapping is undertaken, the Ministry of Health must use existing evidence to initiate appropriate treatment and control activities.

• Podoconiosis: Nationwide mapping integrated with that of LF is the first priority, followed by development of pre- and in-service training modules for school teachers, agricultural extension workers, and all levels of health professionals, in conjunction with the Ministries of Education and Health. Close monitoring and evaluation of community based delivery of prevention and care through inclusion of key indicators into the HMIS will also be important.

• Leprosy: New cases of leprosy are reported at the same rate as decades ago although Ethiopia has reportedly met the elimination target. The difference now is that the country has a much reduced control intensity relying on general health care providers with fewer active leprosy experts linked to control than decades ago. The case of leprosy illustrates the challenges of integrating disease control with adequate vigilance to maintain rather rare excellence accumulated through years of control while adapting to changes in priorities dictated by epidemiologic, socio-economic and development realities.

## Competing interests

The authors declare that they have no competing interests.

## Authors’ contributions

Conceived and designed the review: KD, GD, KD, GD drafted the initial review. KM, TG, AH, AL, AA contributed disease specific information and review. All authors read and approved the final manuscript.
